# Medial gastrocnemius stiffness contributes to active ankle joint stiffness and ground contact time in trained track-and-field athletes

**DOI:** 10.1007/s00421-026-06377-5

**Published:** 2026-08-06

**Authors:** Kazuhiko Yamazaki, Takanori Sugibayashi, Naokazu Miyamoto

**Affiliations:** 1 Faculty of Health and Sports Science, Juntendo University, Chiba, Japan; 2 Faculty of Sport Sciences, Waseda University, 2-579-15 Mikajima, Tokorozawa, Saitama 359-1192 Japan

**Keywords:** Gastrocnemius, Ultrasound, Shear wave elastography, Contact time, Reactive strength index

## Abstract

**Purpose:**

Minimizing ground contact time is important for maximizing stretch–shortening cycle (SSC) performance. However, intrinsic mechanical factors that enable short contact times remain unclear. This study examined whether ankle joint intrinsic stiffness and plantar flexion rate of torque development (RTD) are associated with rebound jump performance, and whether plantar flexor stiffness contributes to these joint-level properties.

**Methods:**

Twenty-four highly trained male track-and-field athletes performed repetitive rebound jumps. Ankle joint intrinsic stiffness was assessed via short-range stretches during isometric plantar flexion at 0%, 20%, and 50% of maximal voluntary contraction (MVC). Medial gastrocnemius (MG) stiffness was measured at the same intensities using shear wave elastography. Plantar flexion RTD was obtained from explosive isometric contractions.

**Results:**

Ground contact time was negatively correlated with active ankle joint stiffness at 50% MVC (r =  − 0.576), and this association remained significant after false discovery rate correction (P = 0.027). Ankle joint stiffness at 20% and 50% MVC was in turn positively correlated with MG muscle stiffness at the corresponding intensities even after false discovery rate correction (20% MVC: r = 0.598, P = 0.027; 50% MVC: r = 0.673, P = 0.009). Plantar flexion RTD was not correlated with any jump variable.

**Conclusion:**

Active intrinsic stiffness of the ankle joint, which is attributable to active MG stiffness, is associated with shorter ground contact time during rebound jumps in trained athletes. These findings suggest that MG stiffness is a mechanical determinant of efficient force transmission during brief ground contact.

**Supplementary Information:**

The online version contains supplementary material available at https://doi.org/10.1007/s00421-026-06377-5.

## Introduction

In stretch–shortening cycle (SSC) actions such as jumping and sprinting, muscle–tendon units undergo rapid lengthening followed by immediate shortening, resulting in enhanced power output (Cavagna 1977; Komi and Bosco 1978). Because this performance-enhancing effect diminishes when the coupling time between the two phases is prolonged (Bosco et al. 1981; Cavagna et al. 1994), minimizing ground contact time is important for maximizing SSC performance. Consistent with this principle, superior sprinters demonstrate shorter ground contact time during sprinting (Morin et al. 2012; Paradisis et al. 2019; Weyand et al. 1985). Despite the importance of short ground contact time, the specific factors that determine an athlete's ability to produce high SSC performance within brief contact periods remain incompletely understood.

From a biomechanical perspective, a stiff ankle joint capable of withstanding high ground reaction forces during the early contact phase likely plays a crucial role in minimizing ground contact time during explosive activities such as jumping and sprinting. Previous research has demonstrated that SSC performance improves with increased stiffness of the plantar flexors and ankle joint during SSC actions (Arampatzis et al. 2001; Stefanyshyn and Nigg 1998). Notably, joint stiffness in SSC actions is determined by both neuromuscular control and the intrinsic mechanical properties of the joint (Bobbert 2001; Hobara et al. 2011). While considerable research has focused on the neuromuscular aspects (Bergmann et al. 2013; Gollhofer and Kyrolainen 1991; Kyrolainen and Komi 1995), less is known about how the intrinsic stiffness of the ankle joint and plantar flexors relates to SSC performance, particularly ground contact time.

However, during sprinting, ground contact time is constrained by running velocity and stride mechanics, which makes it difficult to isolate the contribution of ankle joint properties to contact time per se. Repetitive rebound jumps, by contrast, permit standardized assessment of both jump height and ground contact time, and the reactive strength index (RSI), defined as the ratio of the two, enables separate evaluation of these performance components. Rebound jumps therefore offer a more suitable model for examining how ankle joint intrinsic stiffness and plantar flexion rate of torque development (RTD) each relate to contact time and jump height. Furthermore, whether ankle joint intrinsic stiffness is attributable to the mechanical properties of individual plantar flexor muscles remains unknown. Given that muscle intrinsic stiffness assessed via ultrasound shear wave elastography (SWE) has been associated with various SSC performance metrics, including drop jump height (Ando et al. 2021), repetitive rebound jump height (Ema 2023), and 100-m race time (Miyamoto et al. 2019; Yamazaki et al. 2022), plantar flexor muscle stiffness may be a candidate muscular factor contributing to ankle joint intrinsic stiffness and plantar flexion RTD. Identifying the muscular basis of these joint-level properties would clarify the mechanisms linking muscle properties to SSC performance and inform training approaches aimed at enhancing SSC performance.

Therefore, the present study aimed to examine (1) the associations of ankle joint intrinsic stiffness and plantar flexion RTD with rebound jump performance (ground contact time, jump height, and RSI), and (2) the contribution of plantar flexor muscle stiffness to ankle joint intrinsic stiffness and plantar flexion RTD. We hypothesized that (1) ankle joint intrinsic stiffness would be negatively associated with ground contact time, as a stiffer ankle joint would allow more efficient force transmission during the brief ground contact phase, (2) plantar flexion RTD would be positively associated with jump height, given that rapid torque production is essential for generating sufficient impulse within limited contact periods, and (3) plantar flexor muscle stiffness would contribute to both ankle joint intrinsic stiffness and plantar flexion RTD, thereby indirectly influencing rebound jump performance.

## Methods

### Participants

Twenty-four highly trained male track-and-field athletes specializing in sprinting and jumping events (21.3 ± 1.9 years, 1.75 ± 0.05 m, 68.5 ± 3.8 kg) participated in this study. Inclusion criteria were: (i) current participation in competitive track-and-field events and (ii) a minimum of three years of regular training in sprint or jump disciplines. A priori power analysis using G*Power (Ver. 3.1.9.7, Heinrich-Heine-Universität Düsseldorf, Germany) indicated that a sample size of 23 participants was required to detect a large correlation (|r|= 0.55) with α = 0.05 and statistical power (1 − β) = 0.80, where the expected effect size was based on previous studies reporting associations between ankle joint mechanical properties and SSC performance (Kuitunen et al. 2002; Yamazaki et al. 2022). A sample of 24 athletes was therefore considered adequate for the primary aim of the present study. All participants were informed about the study purpose and experimental procedures before providing written informed consent. This study was approved by the institutional ethics committee, and all procedures followed the Declaration of Helsinki.

### Torque and SWE measurements

**Fig. 1:**
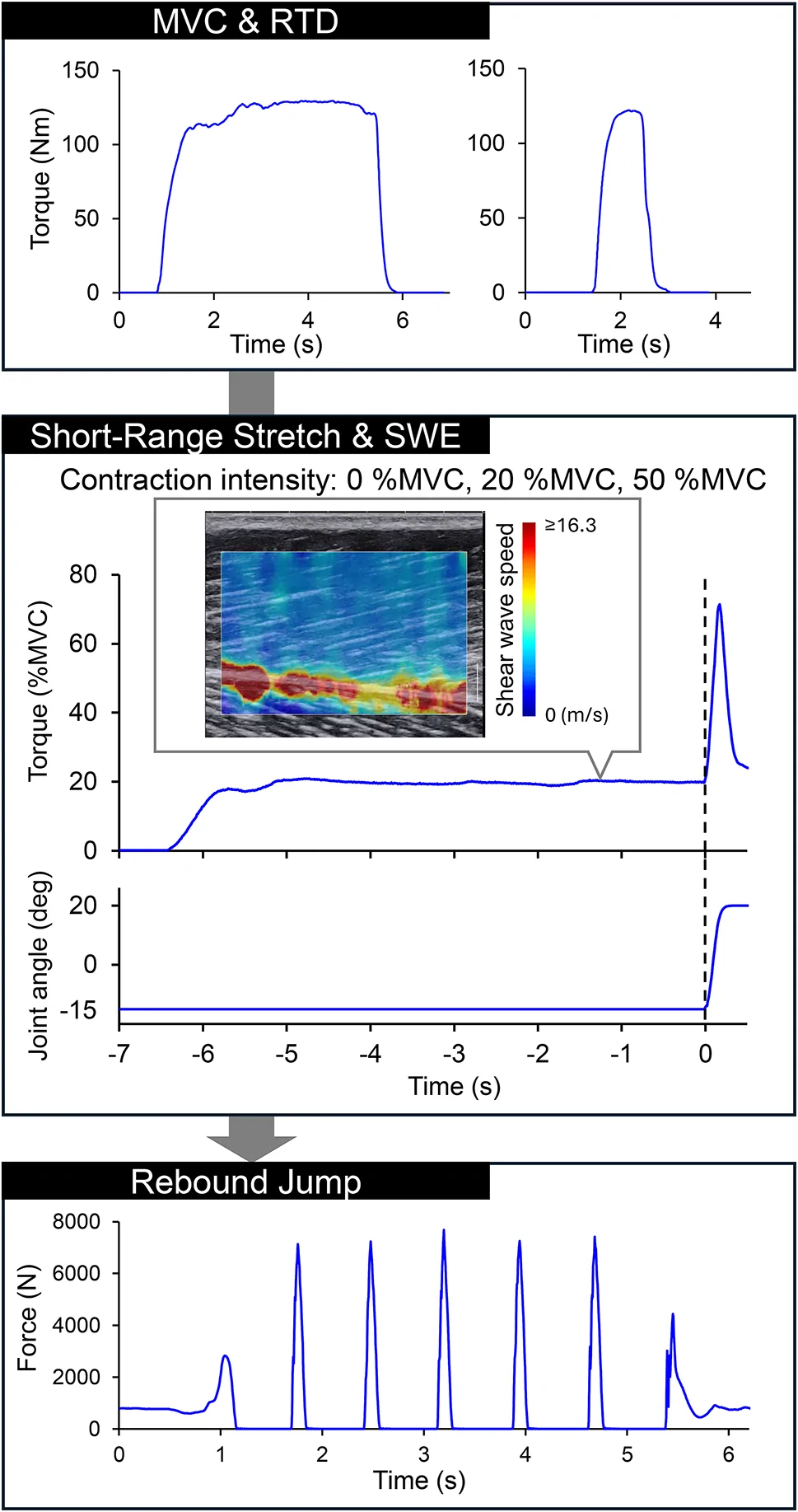
Experimental flow and representative data from one participant. Top panel (MVC & RTD): plantar flexion torque during a maximal voluntary contraction (MVC; left) and an explosive isometric contraction used to determine the rate of torque development (RTD; right). Middle panel (Short-Range Stretch & SWE): plantar flexion torque and ankle joint angle during the short-range stretch protocol, in which a dorsiflexion stretch was applied from − 15° to 20° while the participant maintained the target contraction intensity (0%, 20%, and 50% MVC). The inset shows a representative shear wave elastography (SWE) image of the medial gastrocnemius with the corresponding shear wave speed color scale (0 to ≥ 16.3 m/s). Bottom panel (Rebound Jump): vertical ground reaction force during five repetitive rebound jumps. MVC, maximal voluntary contraction; RTD, rate of torque development; SWE, shear wave elastography

Plantar flexion torque and ankle joint angle were assessed using an isokinetic dynamometer (Biodex System 4, Biodex Medical System, USA) with dedicated software (Research Tool Kit, Biodex Medical System, USA). Participants lay prone on the dynamometer bench with knees fully extended. Their hips and thighs were secured with non-elastic belts to minimize extraneous movement. The right foot was firmly attached to the footplate using non-elastic straps, ensuring that the ankle joint rotational axis was aligned with the footplate axis. This experimental setup and participant positioning were consistent with previously established protocols (Miyamoto and Hirata 2019; Yamazaki et al. 2022). Following a warm-up consisting of submaximal voluntary plantar flexion contractions, each participant performed two isometric maximal voluntary contractions (MVCs) of plantar flexion at an ankle joint angle of -15° (where 0° represents the anatomical neutral position, and positive angles indicate dorsiflexion) (Fig. 1). The slightly plantarflexed position (-15°) was selected for three reasons. First, this position is consistent with the short-range stretch protocol used in previous studies (Miyamoto and Hirata 2019; Yamazaki et al. 2022), which allows direct comparison with these earlier findings. Second, short-range stretch experiments (see below) require a sufficient range of dorsiflexion stretch applied from the starting position toward dorsiflexion. Third, a slightly plantarflexed ankle position approximates the joint configuration adopted during the early ground contact phase of jumping and sprinting (Stefanyshyn and Nigg 1998). The highest MVC torque achieved was used to determine target torque levels (20% and 50% MVC) for subsequent tasks. Subsequently, participants completed five maximal isometric explosive plantar flexion contractions. For each contraction, they were instructed to respond to verbal cues as rapidly and forcefully as possible for approximately 1 s, aiming to exceed 80% MVC torque. Contractions exhibiting baseline torque changes (e.g., countermovement or pretension) exceeding 0.5 N·m before contraction onset were discarded (Miyamoto et al. 2025; Yamazaki et al. 2022).

Following the explosive contraction tasks, short-range stretch experiments were conducted according to established protocols (Foure et al. 2010; Miyamoto and Hirata 2019; Yamazaki et al. 2022). Participants maintained 20% or 50% MVC torque levels (in a randomized order) for approximately 5 s, using real-time visual feedback. During these torque maintenance periods, the medial gastrocnemius (MG) shear wave speed (SWS) was assessed using an ultrasound SWE apparatus (Aixplorer Ver. 12, Supersonic Imagine, France) equipped with a linear array transducer (SL10-2, Supersonic Imagine, France), in “SWE” mode (MSK preset, persistence = off, smoothing = 5) (Fig. 1). Relative torque levels (% MVC) were employed instead of absolute torque (Nm) to standardize muscle contraction intensity across participants and account for individual muscle size differences. A dorsiflexion stretch was then applied using the isokinetic dynamometer, moving the ankle joint from -15° (plantar flexion) to 20° (dorsiflexion), with an angular velocity set at 500°/s. Participants were familiarized with the procedure at each torque level before data collection. Two trials were performed at each torque level, with 1–3 min of rest between trials, extended as necessary. Additional measurements were conducted under passive conditions (0% MVC). For all trials, the ultrasound transducer was positioned at 30% of the lower leg length (measured from the popliteal crease to the lateral malleolus) and aligned with the MG fascicle direction to capture several fascicles without interruption across the B-mode image. Care was taken to avoid muscle compression or deformation during scanning. Three SWE measurements (images) were acquired during each trial. All SWE measurements were performed by an examiner with over 8 years of experience in ultrasound SWE measurements. Torque and ankle joint angle data were sampled at 1 kHz (PowerLab 16/35, ADInstrument, Australia).

Ankle joint stiffness was calculated after correcting passive torque for gravity and inertia effects of the footplate. Ankle joint stiffness was determined as the slope of the torque–angle relationship during the initial 60 ms after stretch onset in each trial (Foure et al. 2010; Miyamoto and Hirata 2019). The average of two trials was used for subsequent analyses. For SWS calculations, SWE images were exported to DICOM format and processed using a custom-written MATLAB script (MathWorks, USA). Mean SWS was calculated over the largest possible region of interest, carefully excluding other tissues (e.g., aponeurosis, subcutaneous fat) and artifacts (void areas). No pixels within the region of interest reached the system’s saturation limit (16.3 m/s). Following previous studies (Bernabei et al. 2020; Kukuchi et al. 2026; Miura and Miyamoto 2025), we reported the squared-value of the SWS as an index of stiffness. Values from three images per trial were averaged, and the mean of two trials was used for further analyses. For explosive contraction tasks, the two best contractions (defined as those with the highest torque at 150 ms after contraction onset) were selected for analysis. RTD was calculated as the slope of the time-torque curve over intervals of 0–150 ms from plantar flexion onset (defined as the instant at which torque exceeded the baseline by 0.5 N·m) following a previous study (Yamazaki et al. 2022). This interval was selected because ground contact time during rebound jumps typically approximates 150 ms (Ema 2023; Zushi et al. 2022). To exclude the effect of MVC torque on RTD, normalized RTD was calculated by dividing the RTD (hereafter referred to as non-normalized RTD) by the MVC torque, and then averaged across the two selected contractions.

### Rebound jump test

A uniaxial force plate (600 mm × 600 mm, PH-6110A, DKH, Japan) was employed for rebound jump testing. Participants were instructed to perform five repetitive rebound jumps, keeping their hands on their hips (akimbo position) as high as possible while minimizing ground contact time. Trials in which hands left the hips were discarded and retested. All participants completed familiarization jumps before data collection. Jump height for each jump was calculated based on flight time using the formula *g* × *t*^2^/8, where *g* is gravitational acceleration and *t* is flight time. RSI was calculated by dividing jump height by ground contact time. Ground contact time was determined by a threshold set as 20 N (Krosshaug et al. 2016; Chun et al. 2023). The average of the two best jumps (defined as those with the highest RSI values) was used for the analysis. Force data were sampled at 1 kHz.

### Statistical analysis

Reliability of the variables measured was evaluated using the within-subject coefficient of variation (CV), intraclass correlation coefficient (ICC[2,1] and 95% confidence interval [CI]), and standard error of measurement (SEM). Relationships between variables were assessed using Pearson's product-moment correlation coefficients. Given the multiple comparisons involved, the Benjamini–Hochberg false discovery rate (FDR) procedure was applied to control for inflated type I error rates, and the results were interpreted primarily on the basis of the FDR-adjusted P-values. To allow readers to compare the two sets of values, both the uncorrected and the FDR-adjusted P-values for the primary analysis are reported in Supplementary Table 1. Effect sizes were interpreted using the following thresholds for correlation coefficients: small (r = 0.10–0.29), moderate (r = 0.30–0.49), and large (r ≥ 0.50) (Cohen 1988). In addition, 95% confidence intervals for correlation coefficients were calculated to provide information on the precision of the estimates. Findings were interpreted based on both statistical significance, judged by the FDR-adjusted P-values, and the magnitude of the effect, judged by the correlation coefficient and its 95% confidence interval. Associations that did not reach significance but showed at least a moderate effect size are therefore discussed as non-significant trends. Data are presented as means ± standard deviations. The significance level was set at α = 0.05. The statistical analyses were performed using statistical software (SPSS Statistics Ver. 30, IBM Japan, Japan).

## Results

**Fig. 2:**
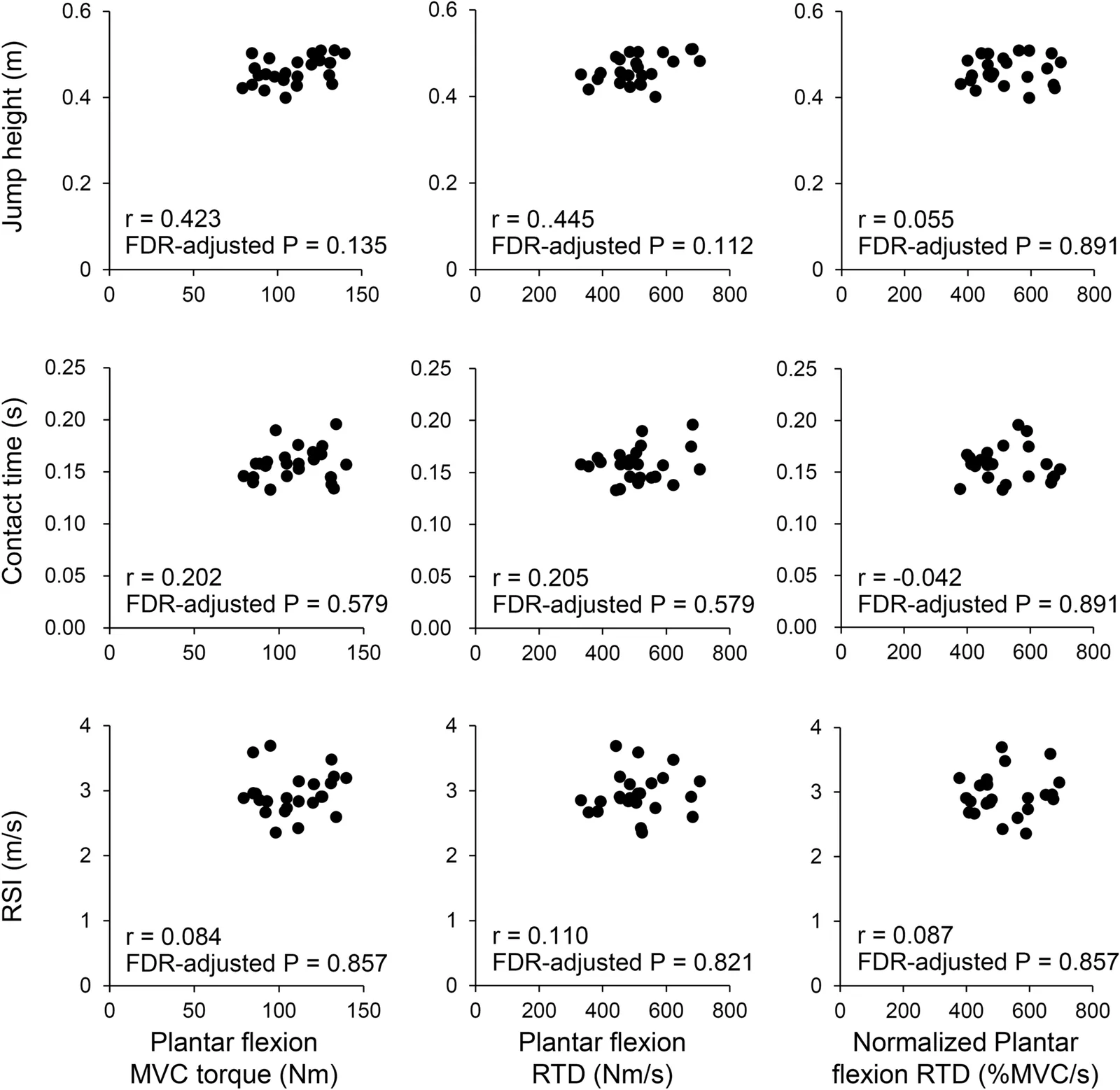
Relationships between rebound jump variables (jump height, contact time, reactive strength index (RSI)) and plantar flexion torque-related variables (maximal voluntary contraction (MVC) torque, non-normalized and normalized rate of torque development (RTD))

The descriptive statistics and reliability for the measured variables are shown in Table 1. Figure 2 shows the relationships between rebound jump variables (jump height, contact time, RSI) and plantar flexion torque-related variables (MVC torque, non-normalized RTD, normalized RTD). Jump height demonstrated a moderate positive correlation with MVC torque (r = 0.423 [95% CI: 0.023–0.706]), although this did not remain significant after FDR correction (FDR-adjusted P = 0.135). No significant correlations were observed between jump height and RTD (non-normalized RTD: r = 0.445 [95% CI: 0.051–0.720], FDR-adjusted P = 0.112; normalized RTD: r = 0.055 [95% CI: -0.356–0.448], FDR-adjusted P = 0.891). Contact time showed no significant correlations with MVC torque (r = 0.202 [95% CI: -0.219–0.560], FDR-adjusted P = 0.579), non-normalized RTD (r = 0.205 [95% CI: -0.217–0.562], FDR-adjusted P = 0.579), or normalized RTD (r = -0.042 [95% CI: -0.438–0.368], FDR-adjusted P = 0.891). RSI showed no significant correlation with MVC torque (r = 0.084 [95% CI: -0.331–0.471], FDR-adjusted P = 0.857), non-normalized RTD (r = 0.110 [95% CI: -0.307–0.492], FDR-adjusted P = 0.821), or normalized RTD (r = 0.087 [95% CI: -0.328–0.474], FDR-adjusted P = 0.857).

**Table 1:** Descriptive and reliability data of measured variables

Variable	Mean ± SD	Min–Max	CV	ICC [95% CI]	SEM
Jump height (m)	0.462 ± 0.032	0.399–0.510	3.9%	0.780 [0.499–0.904]	0.014
Contact time (s)	0.158 ± 0.016	0.133–0.196	3.7%	0.905 [0.784–0.959]	0.003
RSI (m/s)	2.95 ± 0.33	2.36–3.69	5.4%	0.814 [0.576–0.919]	0.121
MVC torque (Nm)	108.6 ± 18.4	79.1–139.7	N.A	N.A	N.A
Normalized RTD (%MVC/s)	521.4 ± 98.5	376.9–693.7	5.8%	0.929 [0.839–0.969]	13.9
Joint stiffness at 0% MVC (Nm/deg)	0.35 ± 0.10	0.24–0.63	2.0%	0.996 [0.990–0.998]	0.001
Joint stiffness at 20% MVC (Nm/deg)	2.65 ± 0.37	2.15–3.59	4.3%	0.926 [0.830–0.968]	0.054
Joint stiffness at 50% MVC (Nm/deg)	4.47 ± 0.50	3.68–5.62	4.8%	0.891 [0.751–0.953]	0.109
MG stiffness at 0% MVC (m^2^/s^2^)	7.1 ± 0.6	5.9–8.3	1.8%	0.949 [0.930–0.987]	0.047
MG stiffness at 20% MVC (m^2^/s^2^)	35.9 ± 9.0	18.0–52.1	3.0%	0.991 [0.978–0.996]	0.170
MG stiffness at 50% MVC (m^2^/s^2^)	72.5 ± 15.6	41.9–104.4	4.4%	0.975 [0.942–0.989]	0.798

*CV* Coefficient of variation, *ICC* Intraclass correlation coefficient, *MG* Medial gastrocnemius, *MVC* Maximal voluntary contraction, *RSI* reactive strength index, *RTD* Rate of torque development, *SEM* Standard error of measurement

Values are presented as the mean of the two selected trials or jumps for all variables, except for MVC torque, for which the higher of the two trials was used. For the rebound jump variables (jump height, contact time, and RSI), the two jumps with the highest RSI values were selected. Reliability indices (CV, ICC, and SEM) were calculated from the two trials or jumps and are expressed in the same units as the corresponding variable. ICC was calculated as ICC(2,1). Reliability indices were not calculated for MVC torque (N.A.), because the higher of the two trials, rather than their average, was used for this variable

**Fig. 3:**
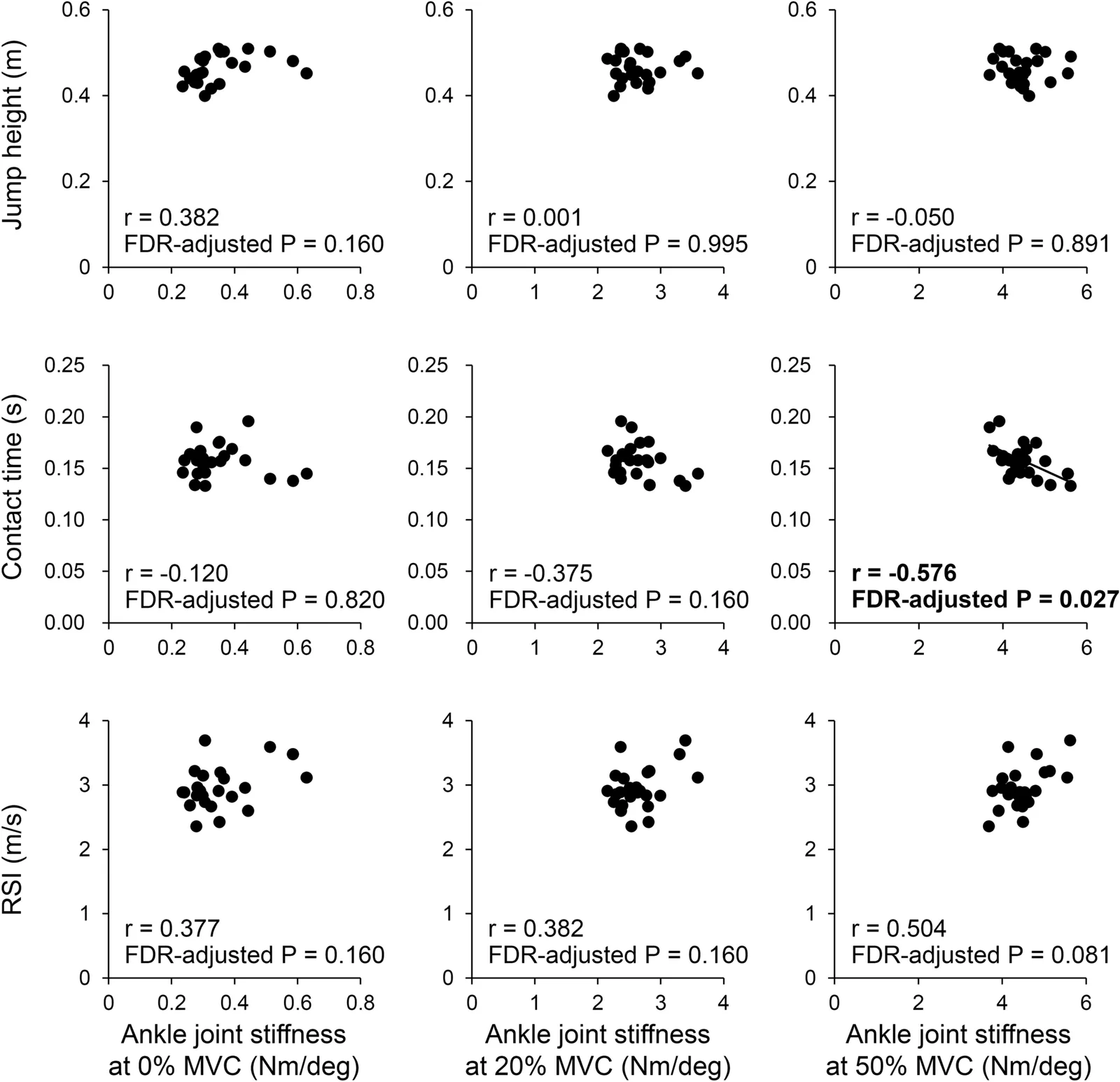
Relationships between rebound jump variables (jump height, contact time, RSI) and ankle joint stiffness at 0%, 20%, and 50% MVC

Figure 3 shows the relationships between rebound jump variables (jump height, contact time, RSI) and ankle joint stiffness at 0%, 20%, and 50% MVC. No significant correlations were observed between jump height and ankle joint stiffness at any contraction intensity (0% MVC: r = 0.382 [95% CI: -0.025–0.681], FDR-adjusted P = 0.160; 20% MVC: r = 0.001 [95% CI: -0.402–0.405], FDR-adjusted P = 0.995; 50% MVC: r = -0.050 [95% CI: -0.445–0.360], FDR-adjusted P = 0.891). Contact time showed no significant correlations with ankle joint stiffness at 0% MVC (r = -0.120 [95% CI: -0.499–0.298], FDR-adjusted P = 0.820) and a moderate negative correlation with ankle joint stiffness at 20% MVC (r = -0.375 [95% CI: -0.676–0.033]), although this did not reach statistical significance (FDR-adjusted P = 0.160). A significant negative correlation was observed between contact time and ankle joint stiffness at 50% MVC (r = -0.576 [95% CI: -0.795–-0.225], FDR-adjusted P = 0.027). RSI showed moderate positive correlations with ankle joint stiffness at 0% MVC (r = 0.377 [95% CI: -0.031–0.678]) and 20% MVC (r = 0.382 [95% CI: -0.026–0.680]), although these did not reach statistical significance (FDR-adjusted P = 0.160 and FDR-adjusted P = 0.160, respectively). RSI showed a moderate positive correlation with ankle joint stiffness at 50% MVC (r = 0.504 [95% CI: 0.126–0.754]), although this did not remain statistically significant after FDR correction (FDR-adjusted P = 0.081).

**Fig. 4:**
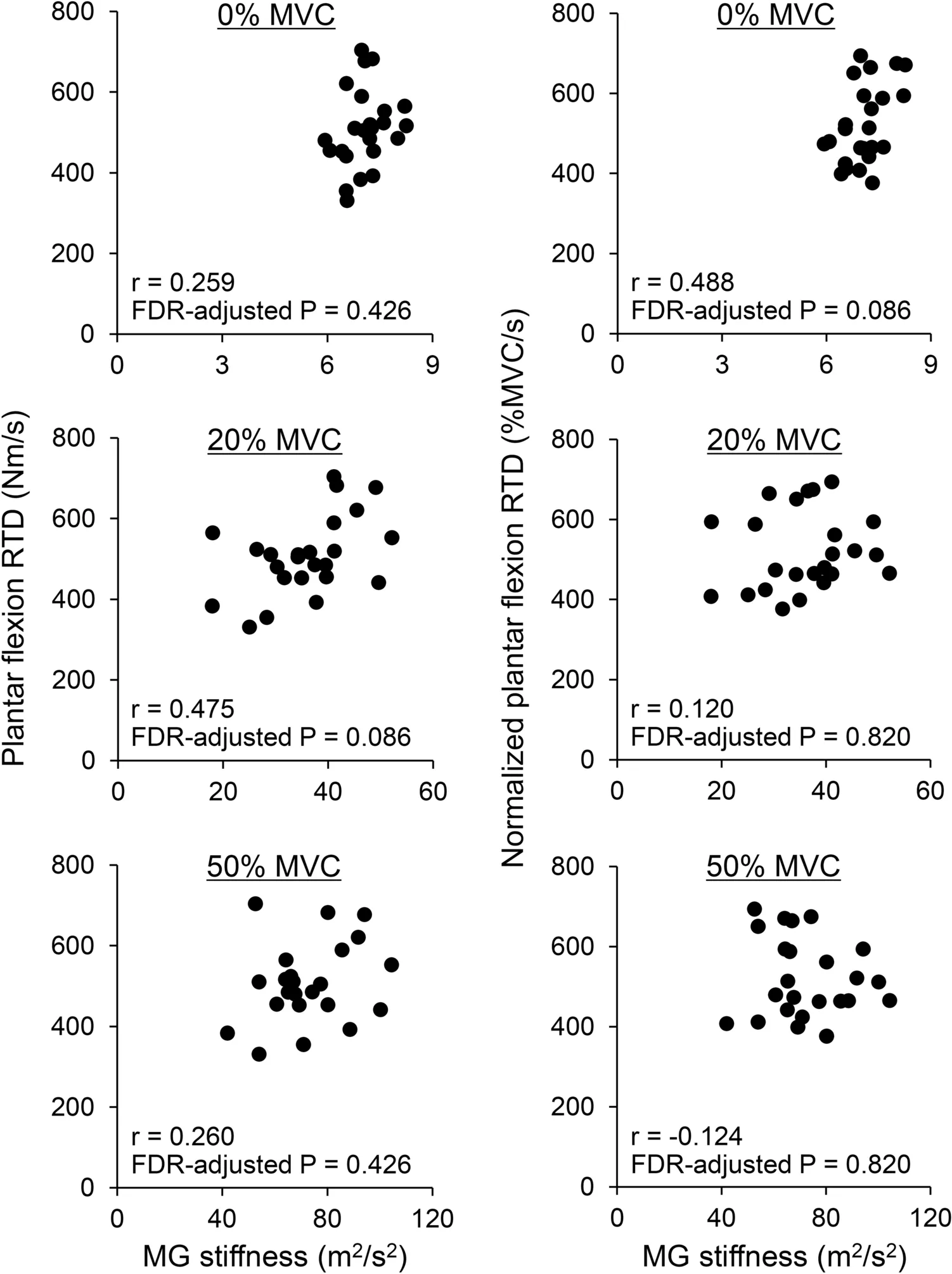
Relationships between RTD (non-normalized and normalized) and MG stiffness at 0%, 20%, and 50% MVC

Figure 4 illustrates the relationships between RTD and MG stiffness at 0%, 20%, and 50% MVC. Non-normalized RTD was positively correlated with MG stiffness at 20% MVC with a moderate effect size (r = 0.475 [95% CI: 0.089–0.737]); however, this association did not remain statistically significant after FDR correction (FDR-adjusted P = 0.086). No such associations were observed for MG stiffness at 0% MVC (r = 0.259 [95% CI: -0.161–0.600], FDR-adjusted P = 0.426) or 50% MVC (r = 0.260 [95% CI: -0.160–0.601], FDR-adjusted P = 0.426). Normalized RTD was positively correlated with MG stiffness at 0% MVC with a moderate effect size (r = 0.488 [95% CI: 0.105–0.745]); however, this association did not remain statistically significant after FDR correction (FDR-adjusted P = 0.086). No such associations were observed for MG stiffness at 20% MVC (r = 0.120 [95% CI: -0.298–0.499], FDR-adjusted P = 0.820) or 50% MVC (r = -0.124 [95% CI: -0.502–0.294], FDR-adjusted P = 0.820).

**Fig. 5:**
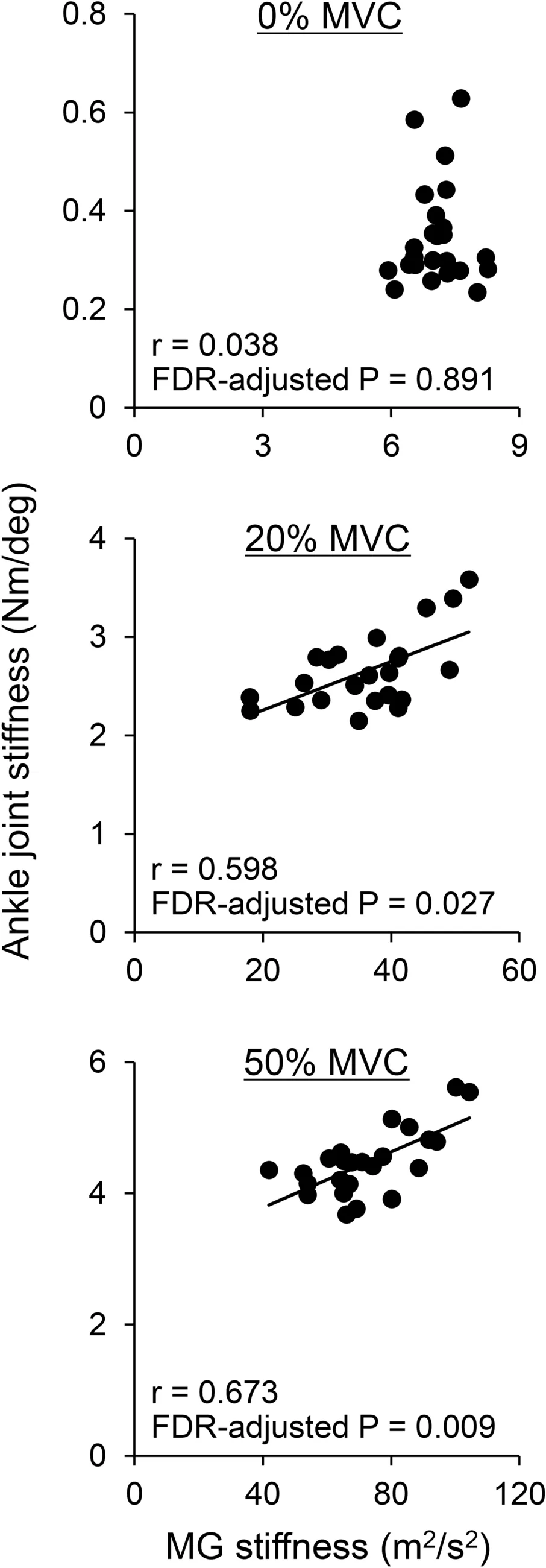
Relationships between ankle joint stiffness and MG stiffness at corresponding contraction intensities

Figure 5 presents the relationships between ankle joint stiffness and MG stiffness at corresponding contraction intensities (0%, 20%, and 50% MVC). Ankle joint stiffness exhibited significant positive correlations with MG stiffness at matching contraction levels of 20% MVC (r = 0.598 [95% CI: 0.256–0.807], FDR-adjusted P = 0.027) and 50% MVC (r = 0.673 [95% CI: 0.370–0.847], FDR-adjusted P = 0.009), but demonstrated no significant correlation at 0% MVC (r = 0.038 [95% CI: -0.371–0.435], FDR-adjusted P = 0.891).

## Discussion

The main findings of this study were: (1) ground contact time during rebound jumps was negatively correlated with active ankle joint intrinsic stiffness at 50% MVC, and (2) active ankle joint intrinsic stiffness was positively correlated with MG stiffness at corresponding contraction intensities of 20% and 50% MVC. These associations remained statistically significant after FDR correction. Furthermore, moderate associations were observed between jump height and MVC torque, between RSI and ankle joint intrinsic stiffness at 50% MVC, and between normalized RTD and passive MG stiffness.

Ground contact time during rebound jumps negatively correlated with active ankle joint intrinsic stiffness in the present study. This association was statistically significant at 50% MVC (r = -0.576, FDR-adjusted P = 0.027) but not at 20% MVC (r = -0.376, FDR-adjusted P = 0.160). Two factors may explain this intensity-dependent result. First, during the early ground contact phase of rebound or drop jumps, the plantar flexor muscles generate forces that considerably exceed 20% MVC to counteract the large dorsiflexion moments produced by ground reaction forces (Hobara et al. 2010; Torres-Banduc et al. 2021). Ankle joint intrinsic stiffness assessed at higher contraction intensities is therefore more representative of the mechanical conditions during actual SSC tasks. Second, ankle joint stiffness at low-to-moderate contraction intensities may be influenced by passive structures such as ligaments and the joint capsule. At higher contraction intensities, the relative contribution of active muscle stiffness increases, potentially amplifying individual differences in joint stiffness and strengthening the association with contact time. Consistent with this reasoning, MG muscle stiffness correlated significantly with ankle joint stiffness at 20% and 50% MVC but not at 0% MVC, indicating that the contribution of active muscle properties to joint stiffness becomes more prominent at higher contraction levels. These findings extend the work of Kuitunen et al. (2002), who reported that ankle joint stiffness during sprint running showed strong negative correlations with contact time (r = -0.81 at 70% running speed to r = -0.92 at maximum speed). However, ankle joint stiffness in their study and in other previous investigations (Hobara et al. 2011; Kuitunen et al. 2011) was derived from kinetic and kinematic data during SSC exercises, meaning that the observed stiffness reflected both neuromuscular control (Hobara et al. 2010) and intrinsic mechanical properties of the muscle–tendon unit. In the present study, we assessed ankle joint intrinsic stiffness under well-controlled isometric conditions using an isokinetic dynamometer (Foure et al. 2010; Yamazaki et al. 2022), thereby excluding confounding factors such as individual differences in joint angles and neuromuscular activation levels during dynamic tasks. The significant correlation between MG muscle stiffness and ankle joint stiffness at matching contraction intensities further confirms that the joint stiffness assessed in our protocol reflects the intrinsic mechanical properties of the plantar flexor muscles rather than neuromuscular modulation alone. Collectively, our findings suggest that intrinsic ankle joint stiffness, attributable to active muscle stiffness of the plantar flexors, plays an important role in determining ground contact time during SSC exercises. This interpretation is supported by Yamazaki et al. (2022), who found that active ankle joint intrinsic stiffness significantly correlated with 100-m sprint performance. Based on our findings, this relationship can be explained by the role of ankle joint intrinsic stiffness in minimizing ground contact time, a critical determinant of sprint velocity (Mattes et al. 2021; Morin et al. 2012).

Contrary to our hypothesis, normalized plantar flexion RTD showed no significant correlations with any rebound jump performance variable. Although the correlation between jump height and MVC torque did not reach significance after FDR correction (r = 0.423), the moderate effect size suggests that maximal strength capacity may contribute to jump height during rebound jumps. The absence of a significant RTD-performance relationship may be explained by the task-specific time constraints of rebound jumps. Ground contact time during rebound jumps averaged approximately 160 ms in the present study, which is longer than that during maximal sprinting (80–110 ms) (Morin et al. 2012; Weyand et al. 1985). This difference is important because RTD is expected to be a limiting factor for performance primarily when the available time for force production is highly constrained. During rebound jumps, the contact period may be long enough for athletes to approach their maximal force levels, making peak strength capacity more relevant than the rate at which force is developed. To examine whether the choice of the 0–150 ms interval underestimated the contribution of RTD, we additionally calculated RTD over the shorter 0–100 ms interval (RTD_100_) and examined its correlations, both non-normalized and normalized, with the rebound jump variables. When the FDR procedure was re-applied with RTD replaced by RTD_100_, the combinations that yielded significant correlations remained identical to those obtained with RTD, and RTD_100_ was not significantly correlated with any rebound jump variable. This result indicates that the absence of a significant association between RTD and rebound jump variables is not attributable to the choice of calculation interval, but rather to the relatively long ground contact time of rebound jumps (approximately 160 ms) compared with maximal sprinting. By contrast, Yamazaki et al. (2022) reported a significant correlation between plantar flexion RTD and 100-m sprint performance, where ground contact time is considerably shorter. These findings collectively suggest that the relative importance of RTD versus maximal strength for SSC performance depends on the duration of ground contact. Importantly, this does not diminish the significance of short contact time in rebound jumps. Even at approximately 160 ms, contact time was significantly associated with ankle joint intrinsic stiffness at 50% MVC, indicating that the ability to transmit force efficiently through a stiff ankle joint remains critical for rebound jump performance regardless of whether RTD per se is the limiting factor. Beyond these task-specific time constraints, two further factors may contribute to the absence of an association between RTD and rebound jump performance. First, rebound jumps involve a rapid eccentric-to-concentric transition, during which elastic energy is stored in and recoiled from the muscle–tendon unit, whereas the explosive isometric RTD test does not include such a stretch–shortening cycle. The rate of force development measured under purely isometric conditions may therefore not reflect the capacity to use stored elastic energy during rebound jumps. Second, rebound jumps are multi-joint movements involving the hip, knee, and ankle, whereas our RTD measurement was restricted to isometric plantar flexion at the ankle. The contribution of other muscle groups, such as the hip and thigh musculature, to rebound jump performance may therefore reduce the likelihood of detecting a relationship with ankle plantar flexor RTD alone.

A moderate positive association was observed between normalized RTD and passive MG muscle stiffness (r = 0.488), which is consistent with previous findings (Ando et al. 2021; Miyamoto et al. 2025; Yamazaki et al. 2022). This relationship aligns with the established role of the extracellular matrix (ECM) in lateral force transmission from muscle fibers (Patel and Lieber 1997), as the ECM is a primary determinant of passive muscle stiffness (Gillies and Lieber 2011; Purslow 1989). Although RTD did not directly correlate with rebound jump performance in the present study, passive muscle stiffness may contribute to rapid force generation capacity, which could be advantageous in SSC tasks requiring shorter contact times than those observed in rebound jumps. In contrast to passive muscle stiffness, active muscle stiffness at 20% and 50% MVC correlated with ankle joint stiffness at corresponding contraction intensities, which in turn was associated with shorter contact time. Ankle joint stiffness also showed a moderate positive association with RSI (r = 0.504). These findings suggest that both passive and active muscle stiffness may play different roles in SSC performance (Miyamoto et al. 2019; Yamazaki et al. 2022): passive muscle stiffness contributes to rapid force development, while active muscle stiffness contributes to efficient force transmission during ground contact. Consequently, augmenting both passive and active muscle stiffness could be beneficial for enhancing SSC performance. However, effective methods for increasing muscle stiffness remain largely unknown. While athletes commonly perform plyometric training with the goal of increasing muscle and tendon stiffness, drop jump training has paradoxically been shown to decrease passive muscle stiffness (Ando et al. 2021), without altering tendon stiffness (Kubo et al. 2020). Interestingly, passive muscle stiffness increased during periods of training cessation in athletes who regularly performed eccentric and plyometric training (Miyamoto et al. 2024). This increase in passive muscle stiffness may be one mechanism underlying performance improvements associated with tapering. Furthermore, a recent study in untrained young individuals reported that long-term collagen peptide supplementation increased passive muscle stiffness, accompanied by improvements in RTD (Miyamoto et al. 2025). Nevertheless, the optimal duration of training cessation for muscle stiffness enhancement, the efficacy of collagen peptide supplementation in trained athletes, and whether such changes translate to improved SSC performance remain unclear. Further research is warranted to identify effective methods for modulating passive and active muscle stiffness and to elucidate their functional significance for athletic performance.

This study has several limitations that warrant consideration. First, we cannot determine the causal relationships between the variables examined, because of the cross-sectional design of the present study. Longitudinal investigations are necessary to establish causality. Second, muscle stiffness measurements were limited to the MG only. Previous research has demonstrated associations between passive muscle stiffness in other muscle groups and athletic performance. For instance, passive muscle stiffness of the vastus lateralis correlates with 100 m sprint performance (Miyamoto et al. 2019) and rebound jump height (Ema 2023). Therefore, expanding the investigation to include muscles of the hip and thigh regions may provide clearer insights into the relationships between muscle stiffness, RTD, joint intrinsic stiffness, and rebound jump performance variables. Third, the direction of the force vectors differs between sprinting and rebound jumps, with sprinting involving larger horizontal force components. Therefore, the present findings, obtained from a predominantly vertical stretch–shortening cycle task, should be generalized to sprinting with caution.

In conclusion, the present study demonstrated that active ankle joint intrinsic stiffness was negatively associated with ground contact time during rebound jumps in trained track-and-field athletes, with a parallel trend toward a positive association with RSI. Furthermore, active ankle joint intrinsic stiffness was positively correlated with active MG stiffness at corresponding contraction intensities, indicating that intrinsic muscle stiffness contributes to ankle joint stiffness during active contractions. Jump height showed a moderate association with plantar flexion MVC torque, suggesting that maximal strength capacity may be relevant for achieving greater jump height. These results indicate that active MG stiffness contributes to active ankle joint intrinsic stiffness, which in turn relates to shorter ground contact time during SSC exercises. This muscle-to-joint-to-performance linkage provides a mechanical framework that may inform future training strategies targeting plantar flexor stiffness for SSC performance enhancement.

## Supplementary Information

Below is the link to the electronic supplementary material.

Supplementary file1 (DOCX 27 KB)

## Data Availability

The datasets generated and/or analyzed during the current study are available from the corresponding author upon reasonable request.

## Abbreviations

ECM: Extracellular matrix
FDR: False discovery rate
MG: Medial gastrocnemius
MVC: Maximal voluntary contraction
RTD: Rate of torque development
SSC: Stretch-shortening cycle
SWE: Shear wave elastography
SWS: Shear wave speed
