# Supplementary material for: Medial gastrocnemius stiffness contributes to active ankle joint stiffness and ground contact time in trained track-and-field athletes
Source: Eur J Appl Physiol. 2026 Aug 6;126(11):6201–12. doi: 10.1007/s00421-026-06377-5 (PMC13751700; doi:10.1007/s00421-026-06377-5)
Supplement: Supplementary file 1 [file 421_2026_6377_MOESM1_ESM.docx]

**Supplementary Table 1. Correlation coefficients with uncorrected and FDR-adjusted P-values for the primary analysis**

|  | r | Uncorrected P-value | FDR-adjusted P-value |
| --- | --- | --- | --- |
| Jump height vs. MVC torque | 0.423 | 0.040 | 0.135 |
| Jump height vs. RTD | 0.445 | 0.029 | 0.112 |
| Jump height vs. Normalized RTD | 0.055 | 0.798 | 0.891 |
| Jump height vs. Joint stiffness at 0%MVC | 0.382 | 0.065 | 0.160 |
| Jump height vs. Joint stiffness at 20%MVC | 0.001 | 0.995 | 0.995 |
| Jump height vs. Joint stiffness at 50%MVC | -0.050 | 0.815 | 0.891 |
| Contact time vs. MVC torque | 0.202 | 0.343 | 0.579 |
| Contact Time vs. RTD | 0.205 | 0.337 | 0.579 |
| Contact Time vs. Normalized RTD | -0.042 | 0.846 | 0.891 |
| Contact Time vs. Joint stiffness at 0%MVC | -0.120 | 0.577 | 0.820 |
| Contact Time vs. Joint stiffness at 20%MVC | -0.375 | 0.071 | 0.160 |
| Contact Time vs. Joint stiffness at 50%MVC | -0.576 | 0.003 | 0.027 |
| RSI vs. MVC torque | 0.084 | 0.698 | 0.857 |
| RSI vs. RTD | 0.110 | 0.608 | 0.821 |
| RSI vs. Normalized RTD | 0.087 | 0.685 | 0.857 |
| RSI vs. Joint stiffness at 0%MVC | 0.377 | 0.069 | 0.160 |
| RSI vs. Joint stiffness at 20%MVC | 0.382 | 0.066 | 0.160 |
| RSI vs. Joint stiffness at 50%MVC | 0.504 | 0.012 | 0.081 |
| RTD vs. MG stiffness at 0%MVC | 0.259 | 0.221 | 0.426 |
| RTD vs. MG stiffness at 20%MVC | 0.475 | 0.019 | 0.086 |
| RTD vs. MG stiffness at 50%MVC | 0.260 | 0.219 | 0.426 |
| Normalized RTD vs. MG stiffness at 0%MVC | 0.488 | 0.016 | 0.086 |
| Normalized RTD vs. MG stiffness at 20%MVC | 0.120 | 0.575 | 0.820 |
| Normalized RTD vs. MG stiffness at 50%MVC | -0.124 | 0.563 | 0.820 |
| Joint stiffness at 0%MVC vs. MG stiffness at 0%MVC | 0.038 | 0.858 | 0.891 |
| Joint stiffness at 20%MVC vs. MG stiffness at 20%MVC | 0.598 | 0.002 | 0.027 |
| Joint stiffness at 50%MVC vs. MG stiffness at 50%MVC | 0.673 | 0.000 | 0.009 |

FDR: false discovery rate, MG: medial gastrocnemius, MVC: maximal voluntary contraction, RSI: reactive strength index, RTD: rate of torque development.
